# Quality of care in university hospitals in Saudi Arabia: an updated systematic review

**DOI:** 10.3389/frhs.2025.1701062

**Published:** 2026-01-15

**Authors:** Abdullah Alalawi

**Affiliations:** Al Qunfudah Health Sciences College, Umm Al-Qura University, Makkah, Saudi Arabia

**Keywords:** health system transformation, Institute of Medicine domains, quality of care, Saudi Arabia, university hospitals, vision 2030

## Abstract

**Background:**

Academic hospitals in Saudi Arabia are major providers of specialised healthcare and serve as training centres for future health professionals. With ongoing reforms under Vision 2030, evaluating the quality of care in these institutions is essential. Despite their strategic importance, no recent systematic review has synthesised evidence on their performance.

**Aim:**

The aim of this systematic review is to identify major issues, barriers, and challenges impacting the quality of care in Saudi university-affiliated hospitals and provide evidence-based recommendations for improvement.

**Methods:**

This systematic review followed PRISMA 2020 guidelines. PubMed, Scopus, Web of Science, Embase, and the Saudi Digital Library were searched for empirical studies published between January 2015 and June 2025—a timeframe chosen to update the last available review, which covered literature up to early 2015, and to capture evidence emerging during major healthcare reforms associated with Saudi Vision 2030. Eligible studies included quantitative, qualitative, or mixed-methods research examining any of the Institute of Medicine quality domains in Saudi university-affiliated hospitals. Data were extracted using a standardised form, and study quality was assessed using the Newcastle–Ottawa Scale adapted for cross-sectional studies. Due to heterogeneity across study designs and outcomes, findings were synthesised narratively.

**Results:**

Twenty-eight studies were included. Patient-centredness was most frequently assessed, showing high satisfaction with communication, respect, and clinician interactions, although waiting times and referral delays were common barriers. Effectiveness was evident in paediatric care and pain management, while chronic disease and rehabilitation outcomes were less favourable. Innovative models such as telemedicine and hypofractionated radiotherapy improved both effectiveness and efficiency. Timeliness challenges were identified in emergency and discharge processes, whereas digital health supported faster access. Efficiency concerns included overcrowding and workflow delays, offset by alternative care models. Safety issues included medication errors, infection control gaps, and punitive cultures, though improvements in teamwork and organisational learning were noted. Equity was least studied, with disparities linked to demographics and geography. Most studies were of moderate quality; six were rated high.

**Conclusion:**

Saudi university hospitals demonstrate strengths in patient-centredness, effectiveness, and efficiency, but persistent gaps in timeliness, safety, and equity remain. Targeted improvements are needed to strengthen their role in advancing healthcare quality and achieving Vision 2030 goals.

## 1 Introduction

Over the past few decades, the Kingdom of Saudi Arabia (KSA) has witnessed significant improvements in access to healthcare services, driven by rapid development, population growth, and substantial government investment in the health sector. However, expanding access has introduced a range of challenges, particularly related to the quality and efficiency of care delivery across institutions (1, 2). Recent analyses of the Saudi health system and financing reforms indicate that, while initiatives such as the Cooperative Health Insurance System have contributed to broader coverage and progress towards universal health coverage, important gaps in service quality, equity, and financial protection persist (3). Moreover, a systematic review of healthcare quality from the perspective of patients in Gulf Cooperation Council (GCC) countries reported mixed levels of satisfaction and ongoing concerns regarding access, communication, and continuity of care, underscoring the need to enhance care quality alongside service expansion (2). These challenges are compounded by increasing healthcare demand, rising operational costs, evolving disease patterns, shortages of healthcare professionals, and system inefficiencies that become particularly visible during periods of high patient volume, such as the Hajj season (3).

Improving the quality of care has therefore become a strategic priority under Saudi Arabia's Vision 2030 health transformation agenda, which emphasises enhanced performance, patient-centredness, and improved patient experience across all healthcare sectors. Recent Saudi-based studies (4, 5) reinforce this focus. For example, Alsubahi et al. (4) found that patient-centred communication, emotional support, and family involvement were strong predictors of better patient-reported outcomes, experiences, and overall satisfaction among individuals with diabetes, highlighting the central role of patient-centred care (PCC) in quality improvement. Similarly, a national study (5) conducted across 47 primary healthcare centres demonstrated that key PCC dimensions, such as access to care, continuity and transition, physical comfort, and information and education, significantly improved patient satisfaction, underscoring the need for health systems to prioritise these domains. Together, these recent findings provide robust evidence that aligns with Vision 2030's mandate to strengthen patient experience and advance high-quality, patient-centred care across Saudi healthcare institutions. To evaluate such improvements, frameworks such as the Donabedian model (structure, process, and outcomes) and the Institute of Medicine's (IOM’s) six domains of quality (safety, effectiveness, patient-centredness, timeliness, efficiency, and equity) are widely used (6).

Academic hospitals, also referred to as university hospitals, serve a dual role in KSA: They provide tertiary-level healthcare services and are affiliated with medical colleges to support education and research (1). Despite their strategic importance, consolidated evidence on how these institutions perform in terms of healthcare quality remains limited. A small number of studies (7–9) have examined aspects such as patient satisfaction, service efficiency, safety practices, and workforce capacity, but no comprehensive synthesis has been conducted to evaluate the overall quality landscape.

In countries such as the United States, academic hospitals are often perceived to offer higher-quality care due to their teaching and research functions (10). Whether this perception holds true in the Saudi context remains unclear. A previous systematic review (11) synthesised early evidence on the quality of care in Saudi university hospitals, identifying challenges such as inconsistent service quality, communication barriers, suboptimal patient-centredness, limited adherence to clinical guidelines, and weaknesses in safety culture. More recently, a systematic review (4) examined healthcare quality from the perspective of patients across GCC countries, including Saudi Arabia, and highlighted persistent issues in patient-centredness, timeliness, diagnostic safety, and equity, further underscoring the need for system-wide quality improvement. However, no review to date has focused specifically on university-affiliated hospitals during the period of major healthcare transformation associated with Saudi Arabia's Vision 2030. This review therefore updates and extends earlier evidence by covering studies published between 2015 and 2025, applying the IOM framework, and examining how recent reforms, governance changes, and service expansions have influenced the quality of care in academic hospitals in KSA. By comparing contemporary findings with earlier literature, this review provides a more nuanced and current assessment of strengths, gaps, and system-level challenges within Saudi university hospitals.

This systematic review aims to provide a comprehensive and up-to-date assessment of the quality of care delivered in university-affiliated hospitals in Saudi Arabia. In particular, the review seeks to identify and categorise the key challenges, barriers, and areas of strength across the six IOM quality domains, drawing on evidence published between 2015 and 2025, a period marked by major healthcare reforms under Saudi Arabia's Vision 2030. By synthesising contemporary findings and comparing them with earlier evidence, including the 2016 systematic review of Saudi university hospitals (11), this review evaluates how quality of care has evolved over time and highlights persistent gaps that require attention. Ultimately, the study aims to generate evidence-based insights to guide policymakers, healthcare leaders, and academic institutions in strengthening quality improvement strategies within the academic hospital sector.

## 2 Materials and methods

### 2.1 Reporting standards and protocol

**Figure 1:**
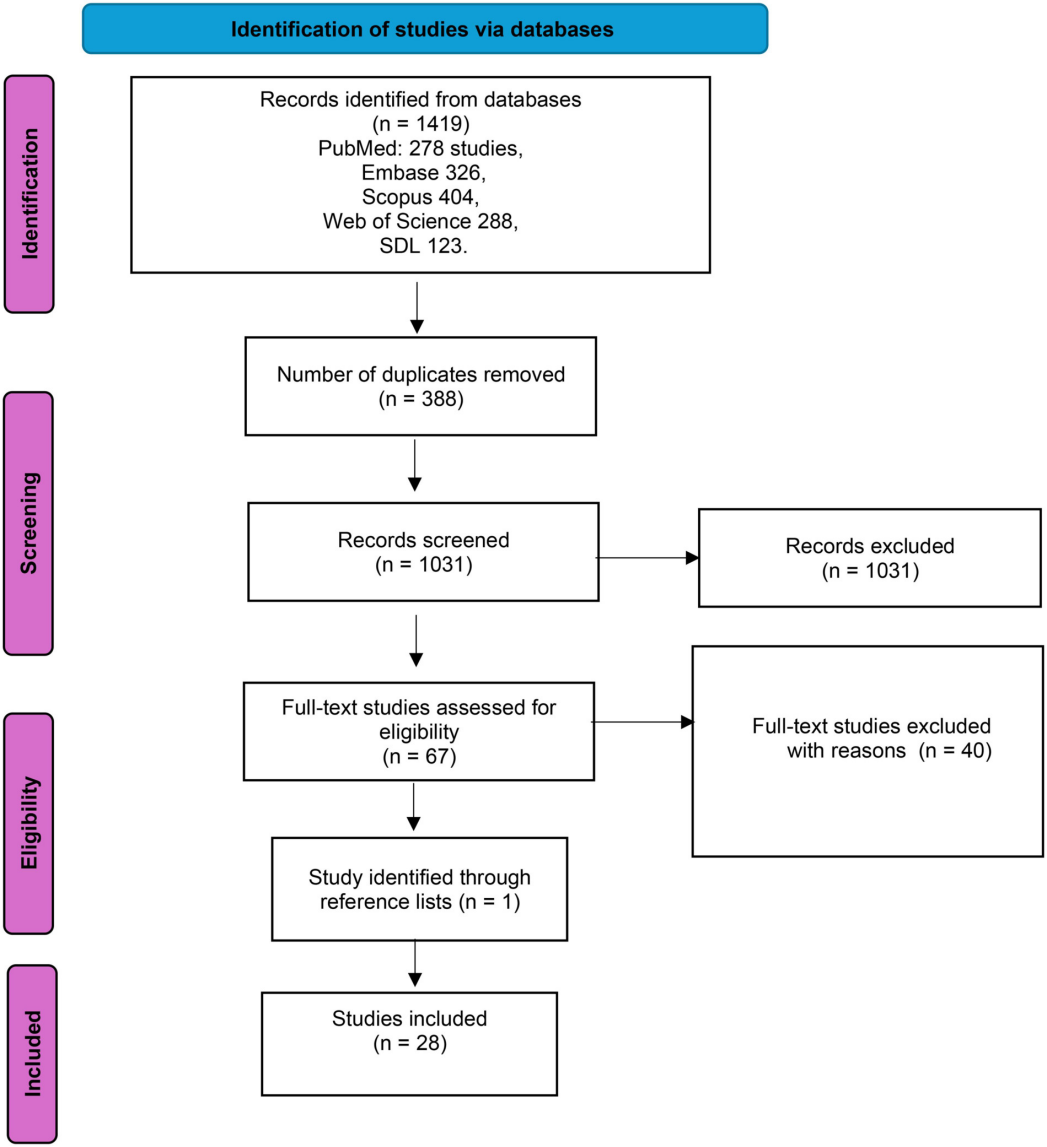
The PRISMA flow diagram (15) was utilised to outline the study selection process in this systematic review.

This systematic review was conducted and reported in accordance with the Preferred Reporting Items for Systematic Reviews and Meta-Analyses (PRISMA) 2020 guidelines (12). The PRISMA flow diagram was used to document the study selection process (Figure 1).

### 2.2 Search strategy

A comprehensive and systematic literature search was undertaken in PubMed, Scopus, Web of Science, Embase, and the Saudi Digital Library to identify relevant studies. The search strategy combined controlled vocabulary terms [e.g., Medical Subject Headings (MeSH) in PubMed and Emtree terms in Embase] with free-text keywords related to healthcare quality, university-affiliated hospitals, and Saudi Arabia. Boolean operators (“AND,” “OR”), truncation symbols, and database-specific field tags were applied to maximise sensitivity and reproducibility.

In PubMed, the search included combinations of “Quality of Health Care” [MeSH], “quality of care,” “healthcare quality,” and “quality improvement” with “University Hospitals” [MeSH], “academic hospital*,” “teaching hospital*,” and “university-affiliated hospital*,” alongside geographic terms for Saudi Arabia. Equivalent strategies were adapted for Scopus, Web of Science, Embase, and the Saudi Digital Library using database-specific syntax. Reference lists of included studies were also screened to identify additional eligible publications.

### 2.3 Eligibility criteria

Studies were included if they met the following criteria: they were empirical studies (quantitative, qualitative, or mixed-methods); examined any aspect of healthcare quality aligned with one or more of the IOM quality domains (safety, effectiveness, patient-centredness, timeliness, efficiency, and equity); were conducted in university-affiliated or academic hospitals located in Saudi Arabia; were published in English or Arabic; and were published between January 2015 and June 2025, a period corresponding to major healthcare reforms under Saudi Arabia's Vision 2030. Studies were excluded if they were editorials, commentaries, conference abstracts, dissertations, or if they were conducted outside academic hospital settings.

### 2.4 Study selection and data extraction

All records retrieved from the database searches were imported into EndNote X9 reference management software, and duplicate entries were removed. Titles and abstracts were initially screened by the first author (AA) to identify potentially relevant studies. Full texts of eligible articles were then assessed against the predefined inclusion criteria. To enhance the reliability of the selection process, an independent reviewer (NA) reviewed a subset of screened records and full-text articles. Any uncertainties or discrepancies regarding study eligibility or data interpretation were resolved through discussion, with further consultation from a senior researcher (MA) when required.

Data extraction was conducted using a standardised extraction form developed for this review. The extracted information included study characteristics, methodological details, outcomes aligned with the IOM quality domains, key findings, and reported limitations. The first author (AA) performed the data extraction, and an independent verification of a subset of extracted data was undertaken by the independent reviewer (NA) to minimise potential errors and interpretation bias. The overall study selection process is illustrated in the PRISMA flow diagram (Figure 1).

### 2.5 Risk of bias appraisal

The methodological quality of the included studies was assessed using the Newcastle–Ottawa Scale (NOS) adapted for cross-sectional studies, as proposed by Herzog et al. (13). Quality appraisal was conducted initially by the first author (AA), who evaluated each study across the three NOS domains: selection of participants (maximum 5 stars), comparability (maximum 2 stars), and outcome assessment (maximum 3 stars), with a total possible score of 10. To enhance consistency and reduce potential bias, an independent reviewer (NA) reviewed a subset of the quality assessments. Any uncertainties or disagreements regarding quality ratings were discussed and resolved through consultation with a senior researcher (MA). Studies scoring 7–10 were considered high quality, 4–6 moderate quality, and ≤3 low quality based on their total NOS scores, and the results of the quality assessment were considered during the interpretation of the findings.

### 2.6 Data synthesis and analysis

Given the heterogeneity of study designs, populations, and outcome measures, a narrative synthesis was undertaken. Findings were analysed thematically and organised using the IOM's six domains of healthcare quality as an analytical framework (14). This framework guided the interpretation and comparison of findings across studies, allowing identification of recurring strengths, challenges, and gaps in the quality of care delivered by university-affiliated hospitals. Results were summarised descriptively and interpreted within the context of Saudi Arabia's healthcare system and ongoing Vision 2030 reforms.

### 2.7 Primary and secondary outcomes

The primary outcome was to identify key challenges, barriers, and issues affecting the quality of care in university hospitals in KSA.

Secondary outcomes included evaluating reported strengths, improvement efforts, and performance across the six IOM quality domains: safety, effectiveness, patient-centredness, timeliness, efficiency, and equity.

## 3 Results

### 3.1 Search results

The literature search was conducted in June 2025 and the study selection process followed the PRISMA 2020 guidelines (15). As illustrated in Figure 1, the initial search identified 1,419 records across five electronic databases: PubMed (*n* = 278), Embase (*n* = 326), Scopus (*n* = 404), Web of Science (*n* = 288), and the Saudi Digital Library (*n* = 123). After removal of 388 duplicate records, 1,031 unique studies remained for title and abstract screening. Of these, 963 records were excluded for failing to meet the inclusion criteria.

A total of 67 full-text articles were assessed for eligibility, of which 40 studies were excluded due to reasons such as being conducted outside university-affiliated hospitals, not assessing healthcare quality domains, or being opinion-based publications. One additional study was identified through reference list screening. Ultimately, 28 studies met all inclusion criteria and were included in the final review.

### 3.2 Study characteristics

The 28 included studies were conducted in major Saudi university-affiliated hospitals across multiple regions. Most studies employed cross-sectional survey designs (16–31), while seven used retrospective observational approaches (32–38). A smaller number of studies adopted prospective (39, 40), repeated cross-sectional (41, 42), or chart review designs (43).

Sample sizes varied considerably, ranging from 41 telemedicine survey respondents (35) to analyses of more than 358,000 emergency department visits (36). Study populations included patients, healthcare professionals (physicians and nurses), hospital administrators, or mixed staff groups. A detailed overview of study characteristics, settings, quality domains assessed, and key findings is provided in Table 1.

**Table 1:** The characteristics of the studies included in this systematic review.

Author(s)	Year	Hospital/setting	Study design	Population/sample size	IOM domains assessed	Key findings	Reported limitations
Abass et al. (16)	2021	King Fahad Medical City, Riyadh	Cross-sectional observational survey (ED-CAHPS)	Patients in Emergency Department/*N* = 266	Patient-centredness, timeliness	High satisfaction with staff communication and cleanliness; long wait times reduced overall satisfaction.	Single-centre study; only ED patients; limited generalisability.
Alhumud et al. (17)	2020	King Abdul-Aziz University Hospital, Endocrinology Clinics, Riyadh	Cross-sectional questionnaire-based study (tele-retinal screening satisfaction)	163 diabetic patients	Patient-centredness (satisfaction), accessibility (timeliness of referral), communication	Overall satisfaction with tele-retinal screening was high (80.4%), particularly for interpersonal manner, general satisfaction, time spent, and communication. Accessibility to the tele-screening itself was also well rated, but satisfaction was lowest for access to ophthalmologists when referral was required. No significant associations were found with patient demographics or diabetes history.	Single-centre study; focused only on endocrinology clinic tele-retinal screening; patients’ concern about referral delays not addressed further.
Alnasser et al. (19)	2020	King Khalid University Hospital (KKUH), Riyadh	Cross-sectional	Outpatients/*N* = 410	Patient safety (safety), communication (patient-centredness)	21.6% did not know drug side effects; 47.8% said physicians did not inform about side effects. 20% reported medical errors; 66.3% did not report errors (54.4% did not know how). 47% misunderstood infection control; 76.5% never asked physicians to wash hands. Older and more educated patients had better knowledge.	Focus on outpatients only; limited to self-reported measures.
Aljaffary et al. (20)	2021	King Fahd University Hospital (KFUH), Imam Abdulrahman Bin Faisal University	Cross-sectional survey (Hospital Survey on Patient Safety Culture, HSOPSC)	900 invited; 805 responded; 600 complete responses (67% response rate)	Patient safety (safety), communication	Strengths: organisational learning 75.4%, feedback/communication about error 67%, frequency of events reported 65.2%. Weaknesses: staffing 20%, teamwork within units 21.4%, non-punitive response to error 21.4%. 42% reported no events; majority rated safety culture excellent/very good.	Single-centre study; cross-sectional; underrepresentation of management and physicians; English survey may limit comprehension; limited generalisability.
Bokhary et al. (18)	2022	KAUH, Jeddah	Cross-sectional questionnaire-based study (Arabic EQS-H scale)	235 patients discharged from medical ward	Patient-centredness (satisfaction), communication, safety (informational needs, privacy)	Overall satisfaction very good (MI) and excellent (RS). Higher satisfaction with planned stay, improved health, higher life satisfaction. Male gender and non-Saudi nationality associated with higher satisfaction.	Single-centre; retrospective phone-based survey; limited generalisability.
Aljadhey et al. (21)	2016	KKUH, Riyadh	Cross-sectional survey (Safety Attitudes Questionnaire)	492 nurses invited; 418 completed (84.9% response rate)	Safety (patient safety culture), teamwork, job satisfaction	Job satisfaction highest (92.7 ± 14.6), working conditions (82.1 ± 16.6), safety climate (75.5 ± 15.5), teamwork climate (75.5 ± 16.7). Low scores in stress recognition (41.9 ± 25.2) and perception of management (68.1 ± 19.1). Female nurses had higher teamwork and working condition scores. More experience correlated with higher job satisfaction and stress recognition.	Single-centre; conducted during accreditation preparation; limited generalisability.
Traiki et al. (41)	2020	KKUH, King Saud University, Riyadh, Saudi Arabia	Retrospective and cross-sectional study (HCAHPS satisfaction survey)	331 surgical patients; 223 completed satisfaction survey	Patient-centredness (satisfaction), safety (surgical outcomes), communication	77.6% nurses treated patients with courtesy/respect: 93% for doctors. 90.3% satisfied with hospital sanitary measures. 64.1% received written discharge instructions. Surgical outcomes: 12% ICU admission, 10.9% complications, 0.9% mortality, 1.8% readmission. 94% would recommend hospital.	Retrospective cross-sectional design; no socio-economic data; single-centre; limited generalisability.
Abualenain et al. (39)	2018	KAUH, Jeddah	Prospective observational study (Point-of-care testing at ED triage)	94 ED patients with high-risk complaints triaged as non-critical	Patient safety (safety of triage decisions), timeliness (faster triage/care)	POCT at triage changed immediate care in 11 cases (12%) and triage level in 12 cases (13%). Triage nurses found POCT helpful in 93% of cases. Most common complaints: chest pain (42%), abdominal pain (31%), shortness of breath (22%).	Single-centre; convenience sample; no follow-up on patients who left without being seen; cost of POCT; subjective triage decisions.
Albarrak et al. (22)	2025	KKUH, Riyadh	Descriptive cross-sectional survey (validated online questionnaire)	412 parents of paediatric patients	Patient-centredness (satisfaction), safety, timeliness, effectiveness, efficiency	Highest satisfaction: Safety 86%, appropriateness 84%, access/timeliness 84%, effectiveness 82%, efficiency 77%. Positive correlation between satisfaction and perception. 49% strongly agreed they could follow doctor's advice; 52.9% strongly agreed doctors treated them with respect.	Self-reported data; single-centre; recruitment via WhatsApp may cause selection bias; limited qualitative insights; limited generalisability.
Almalky et al. (23)	2021	KKUH, Riyadh	Cross-sectional online/telephone survey	141 psychiatric patients followed via telepsychiatry during COVID-19	Patient-centredness (satisfaction), timeliness (access and follow-up), privacy (safety aspect)	Overall satisfaction 94.3%. 80.1% satisfied with structure, 95.7% with process, 96.5% with outcome. Comfort, privacy, easy access, carefulness, and skilfulness of clinicians rated highly. About half would continue using telepsychiatry.	Small sample size: conducted during lockdown may overestimate satisfaction; single-centre study; limited generalisability.
Al-Abbadi et al. (24)	2019	KAUH, Jeddah	Descriptive cross-sectional prospective study	150 elective surgery patients (≥18 years, hospitalised >24 h)	Patient safety, patient-centredness (satisfaction, communication)	Pre-surgery satisfaction M = 8.51, post-surgery satisfaction M = 9.05. 73.3% encouraged to ask questions, 76.7% definite physical comfort, 100% received post-surgery contact. High ratings for communication, education, and safety practices.	Single-centre; elective surgeries only; limited generalisability.
Alswat et al. (42)	2017	King Saud University Medical City (multi-site tertiary teaching hospital), Riyadh	Repeated cross-sectional survey (HSOPSC) comparing 2012 and 2015	4,500 surveys sent: 2,592 respondents (56.7%) across multiple disciplines	Patient safety (safety culture), teamwork, communication, organisational learning	Areas of strength: teamwork within units (84.8%), organisational learning (86.3%), management support for safety (75.3%), feedback and communication about error (71.8%). areas needing improvement: staffing (33.8%), non-punitive response to error (24.8%). improvements from 2012 to 2015 in most composites. better management support linked to higher safety grades and reporting.	Cronbach's alpha low for some composites; multi-language survey may affect consistency; single organisation; limited generalisability.
Albishi et al. (25)	2019	KAUH, Jeddah	Cross-sectional questionnaire-based survey	119 physicians (77.3% interns, 13.4% residents, 9.2% specialists)	Patient safety (knowledge and prevention of surgical site infections)	55.5% Knew definition of SSI; 25.2% knew incidence; 78.2% knew best time for prophylactic antibiotics. Overall knowledge: 6.7% good, 63% fair, 30.2% poor. Mean knowledge score 10.93/20.	Limited to KAUH physicians; findings may not generalise; self-reported survey data.
Aljuaid et al. (34)	2021	King Saud University Medical City, Riyadh	Retrospective analysis of Electronic Occurrence Variance Reporting (E-OVR) system	2,626 reported medication errors	Patient safety (medication error surveillance and patterns)	Prescribing errors most prevalent (55%), followed by availability (14.1%) and delayed medication (10.1%). Medication errors significantly more frequent on weekdays than weekends (*p* = 0.01). On weekends, errors more likely at night shifts (*p* < 0.05). Administration errors least common (0.6%).	Voluntary reporting may underestimate actual incidence; cultural factors may influence reporting.
Aljohaney et al. (31)	2023	King Abdulaziz University Hospital, Jeddah	Cross-sectional observational study using a structured patient satisfaction questionnaire	450 adult outpatients from internal medicine and specialty clinics	Patient-centredness, timeliness, efficiency	• Overall satisfaction score: 4.1/5. • High satisfaction associated with short waiting times, effective physician communication, and clarity of discharge instructions. • Female patients reported slightly higher satisfaction. • Long waiting times negatively affected satisfaction.	• Single-centre study; • Possible response bias due to self-reported measures; • Results may not generalise to inpatient settings or other institutions.
Alnasser et al. (36)	2023	King Abdullah Bin Abdul-Aziz University Hospital	Retrospective observational study using emergency department utilisation data	358,000 ED visits analysed over one year	Efficiency, timeliness, safety	• Majority of ED visits (65%) were non-urgent (Canadian Triage Level 4 or 5). • Significant overcrowding and resource misallocation. • Low re-visitation and admission rates from non-urgent visits. • Recommendations for improved triage and public education to optimise ED use.	• Single-centre study; • Retrospective design limits causality; • Results may not be generalisable to other regions or hospital types.
AlRuthia et al. (32)	2019	King Saud University Medical City, Riyadh	Retrospective cohort study analysing medication prescription and dispensing delays in discharge process	270 medical charts reviewed	Timeliness, efficiency, safety	• Average discharge process time was 5.5 h. • Main delays stemmed from physician order entry and pharmacist verification. • Delays in medication delivery led to prolonged hospital stays. • Recommendations include integrated electronic systems and workflow redesign.	• Single-site study; • Lack of patient outcome tracking; • May is not generalisable to non-teaching hospitals.
Memon (33)	2022	Tertiary care teaching hospital, Riyadh (King Saud University)	Retrospective observational (medical record review	253 patient medical records (General Surgery Dept.)	Safety	98.2% of near-miss incidents were medical errors (mostly prescribing 89.7%, wrong dose/strength 46.6%). Reporting systems (Datix, variance reporting) identified gaps in communication, compliance, and medication safety. Recommended continuous quality improvement and better reporting culture.	Small sample size, single hospital, limited generalisability, missing/incomplete records excluded. Near-miss incidents not fully classified by corrective actions.
Balhaddad et al. (26)	2018	College of Dentistry, Imam Abdulrahman Bin Faisal University, Dammam	Cross-sectional survey (questionnaire, 5-point Likert)	262 patients (out of 320 invited, 81.9% response rate), aged 18–74 years	Patient-centredness, timeliness, efficiency	High overall satisfaction: treatment (mean 4.38), facilities (4.34), appointments (4.27). Older patients (41–74 years) and less educated patients were more satisfied (*p* < 0.05). Quality of treatment and free services were main reasons for attending. Dentist concern and communication scored highest satisfaction.	Convenience sampling (limits generalisability), Likert scale may restrict nuanced responses, single-centre study, limited use of open-ended questions.
Attar et al. (27)	2022	King Abdulaziz University Hospital (KAUH), Jeddah	Cross-sectional survey	60 Psoriatic arthritis patients (78.3% female, mean age 50.3 years)	Patient-centredness, effectiveness, equity	Psoriatic arthritis was associated with markedly reduced quality of life (mean SF-36 = 59.99), with poorer outcomes linked to fatigue, disease activity, older age, and higher BMI. The study concluded that PsA significantly impairs wellbeing and requires targeted interventions to improve QoL.	Small sample size, single tertiary centre, no comparison group, no data on comorbid conditions (e.g., diabetes, HTN), limits generalisability.
Alshareef et al. (35)	2021	King Abdulaziz University Hospital, King Saud University, Riyadh	Retrospective chart review + patient satisfaction survey	339 adult rhinology patients; 41 responded to satisfaction survey	Patient-centredness, timeliness, efficiency, safety	Teleconsultations effectively managed 91.7% of rhinology patients, with only 1.5% requiring in-person visits. Chronic rhinosinusitis with nasal polyposis was the most common diagnosis. Overall satisfaction was high (83.3%), with most patients reporting time and cost savings, though some still preferred in-office visits for clinical assessment.	Exclusively phone-based (no video), retrospective design, limited survey response (41/80 targeted), many patients unreachable (*n* = 154).
Arishi et al. (37)	2022	KKUH, Riyadh	Retrospective cross-sectional study	309 patients prescribed rivaroxaban	Safety, effectiveness	18% of prescriptions were inappropriate, mainly due to wrong dosing/frequency (40% of NVAF, 53% of DVT/PE cases). Some inappropriate co-prescribing with warfarin, aspirin, and clopidogrel. Highlighted risk of adverse drug events and recommended pharmacy-led interventions.	Single-centre, retrospective design, no outcomes tracked beyond prescribing appropriateness, lacks long-term follow-up or multicentre validation
Alzakri et al. (28)	2023	KKUH, Riyadh	Cross-sectional survey	340 orthopaedic patients attending physiotherapy (response rate 84.6%)	Patient-centredness, timeliness, efficiency, effectiveness	Common barriers: ongoing pain (mean = 1.36), distance (1.35), lack of transport (1.33), crowded centres. Facilitators: choice in activities, regular assessments, goal setting, good relationship with providers. Rural residents reported higher barrier and facilitator scores. Concluded that a patient-centred approach, pain management, and regular evaluation improve adherence and outcomes.	Single centre, limited to orthopaedic patients, self-reported survey (risk of response bias), excludes other specialties, findings not generalisable nationwide.
AlThubaity and Shalby (29)	2023	Najran University Hospital and King Khalid Hospital, Najran	Descriptive cross-sectional survey	400 healthcare team members, 150 nurses, 67 physicians, 29 aides, 30 health workers, 124 others)	Safety, effectiveness, efficiency	Five strategies with 28 interventions were identified to reduce nursing errors, mainly targeting falls, medication, documentation, equipment injuries, and missed care. Two-thirds of staff recognised these strategies, and training attendance was linked to higher awareness (*p* < 0.05).	Convenience sampling limits generalisability, single-region study, self-reported perceptions, not outcome-based.
Mulla et al. (38)	2025	King Abdulaziz University Hospital and King Faisal Specialist Hospital and Research Centre, Jeddah	Multicentre retrospective cross-sectional study with patient survey	187 eligible, 153 completed survey (82% response)—breast cancer (*n* = 102), prostate cancer (*n* = 51)	Patient-centredness, timeliness, efficiency, equity, effectiveness	43%–55% travelled from outside Jeddah (avg. 415 km). HFRT saved $101–$133 (BC) and $213–$320 (PC) in travel costs, $367–$1,600 in accommodation, and 9.25–30 h of time. All patients satisfied; 42% preferred shorter stay, 8% financial reasons. 24% BC and 12% PC patients reported mental health strain from daily visits. HFRT improved access, efficiency, and satisfaction.	Retrospective design: reliance on patient recall for costs; limited to Jeddah centres; distances estimated by region not exact address.
AlSadah et al. (40)	2023	King Fahad Hospital of the University, Al Khobar	Prospective cross-sectional survey	200 postoperative patients (18+, ASA 1–3, elective surgeries under GA)	Patient-centredness, timeliness, effectiveness	95.5% overall satisfaction with PACU pain management services. 99.5% reported PACU staff courteous and professional. Pain significantly reduced post-analgesia (*p* < 0.001). Higher satisfaction in older patients (>45), ASA 3, and those with prior surgical history. 86.5% would use same analgesia again, 84.5% recommend to others.	Cross-sectional design (recall bias possible), excludes regional/local anaesthesia and ICU/emergency cases, did not assess surgeon reassurance, single-centre limits generalisability.
Badheeb et al. (43)	2024	King Khalid Hospital, Najran	Retrospective chart review	122 adult ED patients with delayed visits	Timeliness, efficiency, safety	Mean ED stay: 6.1 ± 1.8 h; 54.1% exceeded 6 h. Leading causes of delay: multiple consultations and further investigations (37.7%), team conflict (36.1%). Significant predictors of prolonged stay: holiday visits (*p* < 0.001), CTAS level 4/5 (*p* = 0.003), multiple consultations (OR 2.82, *p* = 0.013), team conflict (OR 2.50, *p* = 0.031). Concluded that better teamwork, monitoring consultation timeframes, and accelerated decision-making could reduce delays.	Single-centre, retrospective, small ample size, conducted during COVID-19 pandemic, limited generalisability.
Alsohime et al. (30)	2019	King Saud University Medical City (KSUMC), Riyadh	Cross-sectional survey	112 paediatric physicians (86% response rate)	Safety, effectiveness, efficiency, patient-centredness	86.6% attended training before EHR launch. Perceived usefulness means 6.4/10; satisfaction 5.2/10. Top usefulness indicator: error reduction and improved quality of care (RII 82.8%). Top satisfaction factor: enhanced individual performance (RII 60.9%). Main barriers: limited hardware availability (RII 38.2%), insufficient IT support, and time-consuming data entry. Concluded that better IT support and hardware availability are essential for improved adoption.	Single-discipline focus (paediatrics only), single hospital, no evaluation of patient-level outcomes, results not generalisable to all specialties.

SSI, surgical site infection; NVAF, non-valvular atrial fibrillation; HTN, hypertension; HFRT, hypofractionated radiotherapy; CTAS, Canadian triage and acuity scale.

To facilitate interpretation and highlight evidence gaps, a summary table was developed to illustrate the distribution of included studies across Saudi regions and the IOM quality domains, highlighting both thematic concentration and areas with limited available evidence (Table 2).

**Table 2:** Distribution of included studies across Saudi regions and IOM quality domains.

Saudi region/IOM domain	Safety	Effectiveness	Patient-centredness	Timeliness	Efficiency	Equity
Riyadh	●●●●●● ●●●●●● ●●	●●●●	●●●●●●●●●	●●●●●●●●	●●●●●●	–
Jeddah	●●●●	●●	●●●●●	●●●	●●	●●
Najran	●●	●	–	●	●●	–
Al Khobar	–	●	●	●	–	–
Dammam	–	–	●	●	●	–

The number of symbols represents the number of included studies.

### 3.3 Findings by IOM domains

Several studies highlighted both strengths and persistent gaps in patient safety. Positive organisational learning and feedback loops were noted (20, 42), while weaknesses included inadequate staffing and punitive error cultures. Medication-related risks were frequently reported, such as prescribing errors and inappropriate anticoagulant dosing (34, 37). Patient-reported safety gaps included poor communication about drug side effects and infection control (19).

#### 3.3.1 Effectiveness

Eight studies examined effectiveness-related outcomes, focusing on treatment outcomes, quality of life, and the impact of different service delivery models. High levels of effectiveness were reported in several clinical services. For example, Albarrak et al. (22) reported high parental satisfaction with paediatric care, with effectiveness rated at over 80%. Similarly, AlSadah et al. (40) found that 95.5% postoperative pain management services achieved high satisfaction levels, with significant reductions in pain following analgesia (*p* < 0.001).

In contrast, studies addressing chronic disease management identified ongoing challenges. Attar et al. (27) demonstrated that psoriatic arthritis substantially impaired health-related quality of life, with disease activity, fatigue, age, and body mass index associated with poorer outcomes. In physiotherapy settings, Alzakri et al. (28) identified barriers such as persistent pain and distance to facilities, while supportive patient–provider relationships and goal setting were associated with improved outcomes.

Several studies evaluated effectiveness through innovative service models. Telemedicine follow-up services were shown to be effective in managing rhinology patients, with a large proportion of cases resolved remotely (35). Likewise, hypofractionated radiotherapy reduced treatment burden and improved access while maintaining high patient satisfaction (38).

Overall, effectiveness-related findings highlighted strong performance in targeted services, alongside persistent barriers in chronic disease management and rehabilitation care.

#### 3.3.2 Patient-centredness

Patient-centredness was one of the most frequently assessed domains. Several cross-sectional surveys highlighted high levels of satisfaction with staff communication, courtesy, and respect. For example, Abass et al. (16) reported strong satisfaction with staff interactions and hospital cleanliness, although long waiting times reduced overall satisfaction. Similarly, another study (18) found generally high satisfaction with planned hospital stay and improved health outcomes, with male and non-Saudi patients expressing comparatively higher satisfaction.

Specific service models further enhanced patient experience. High satisfaction was reported for tele-retinal screening, telepsychiatry, and telemedicine consultations, particularly in relation to privacy, comfort, and convenience (17). Parents of paediatric patients consistently reported high satisfaction with safety, appropriateness of care, and physician respect (22, 23).

However, barriers to patient-centred care were also identified. Long waiting times, distance to facilities, transport limitations, and restricted access to specialists following referral negatively affected patient experiences (28, 35, 41). Despite overall high satisfaction, these findings indicate that patient-centred care delivery remains uneven across services and settings.

#### 3.3.3 Timeliness

Fourteen studies examined timeliness, primarily through waiting times, emergency department length of stay, referral delays, and discharge processes. Prolonged waiting times were repeatedly associated with lower patient satisfaction, particularly in emergency and outpatient settings.

Several studies highlighted delays in discharge processes, with physician order entry and pharmacy verification contributing to prolonged hospital stays (16, 31, 32). Emergency department studies reported extended lengths of stay, often exceeding 6 h, driven by multiple consultations, diagnostic investigations, and workflow inefficiencies.

Conversely, technology-enabled services demonstrated improvements in timeliness. Telemedicine and telepsychiatry services facilitated timely access to care, reduced the need for in-person visits, and maintained high levels of patient satisfaction (23, 35).

#### 3.3.4 Efficiency

Ten included studies examined the IOM domain of efficiency, focusing on resource utilisation, service delivery models, discharge processes, and the use of telemedicine to optimise care delivery.

AlRuthia et al. (32) assessed discharge efficiency and reported a mean discharge process time of 5.5 h, with delays in physician order entry and pharmacist verification contributing to prolonged hospital stays. Similarly, Badheeb et al. (43) identified inefficiencies in emergency department workflows, where more than half of the patients experienced lengths of stay exceeding six hours due to multiple consultations and inter-professional team conflicts.

Inefficient use of emergency services was highlighted by Alnasser et al. (36), who analysed over 358,000 emergency department visits and found that approximately 65% were classified as non-urgent, indicating substantial overcrowding and misallocation of healthcare resources. In addition, Memon (33) reported inefficiencies in surgical safety reporting systems, noting underreporting of near-miss incidents and inconsistent documentation of corrective actions.

Several studies demonstrated efficiency gains through innovative care delivery models. Alshareef et al. (35) found that telemedicine in rhinology significantly reduced unnecessary in-person visits, with 91.7% of patients managed remotely. Similarly, Almalky et al. (23) reported that telepsychiatry services maintained high patient satisfaction while enabling efficient access to care during the COVID-19 pandemic. Mulla et al. (38) further showed that hypofractionated radiotherapy improved efficiency by reducing patient travel, accommodation costs, and overall treatment time, while maintaining high satisfaction levels.

Additional efficiency-related findings were observed in dental and outpatient care settings. Balhaddad et al. (26) reported that free services provided in university dental clinics contributed to high patient satisfaction and perceived efficiency of care. Likewise, Aljohaney et al. (31) demonstrated that shorter waiting times in outpatient departments were associated with improved service efficiency and higher patient satisfaction.

#### 3.3.5 Equity

Two included studies addressed the IOM domain of equity, highlighting disparities in health outcomes and access to specialised services.

Attar et al. (27) examined patients with psoriatic arthritis and found that older age and higher body mass index were significantly associated with poorer health-related quality of life, indicating inequities in outcomes across demographic groups.

Mulla et al. (38) conducted a multicentre retrospective cross-sectional study that included a patient survey among breast and prostate cancer patients. The study reported that 43%–55% of patients travelled from outside Jeddah, with an average distance of 415 km, to access hypofractionated radiotherapy. This treatment model improved equity of access by reducing travel costs (US$101–133 for breast cancer and US$213–320 for prostate cancer), accommodation expenses (US$367–1,600), and overall treatment time (9.25–30 h saved). However, despite these benefits, a proportion of patients reported mental health strain related to frequent treatment visits, including 24% of breast cancer patients and 12% of prostate cancer patients. These findings underscore persistent equity challenges for patients residing far from specialised cancer centres.

#### 3.3.6 Safety

Thirteen included studies examined issues related to the IOM domain of safety, focusing on safety culture, medication practices, surgical outcomes, and technology-supported interventions.

Aljaffary et al. (20) reported strengths in organisational learning (75.4%) and communication about errors (67%) but identified notable weaknesses in staffing levels (20%) and non-punitive responses to errors (21.4%). Similarly, Alswat et al. (42) observed improvements in teamwork and communication about errors over time, while staffing-related challenges persisted.

Medication safety emerged as a recurring concern across several studies. Aljuaid et al. (34) found that prescribing errors accounted for 55% of reported medication errors. In addition, Memon (33) and Arishi et al. (37) highlighted inappropriate prescribing practices and underreporting of near-miss incidents, indicating gaps in medication safety reporting systems. Albishi et al. (25) further reported limited physician knowledge regarding surgical site infection prevention.

In the context of surgical care, Traiki et al. (41) documented adverse outcomes, including intensive care unit admissions (12%), postoperative complications (10.9%), and mortality (0.9%). Studies examining technology-supported interventions suggested potential safety benefits. For example, Abualenain et al. (39) and Alsohime et al. (30) reported that point-of-care testing and electronic health records supported safer clinical practices; however, challenges related to information technology infrastructure and workflow integration remained.

### 3.4 Risk of bias appraisal

Using the Newcastle–Ottawa Scale (NOS) adapted for cross-sectional studies, the methodological quality of the 28 included studies was assessed across the domains of selection, comparability, and outcome. Overall, six studies (20, 22, 31, 36, 38, 42) were rated as high quality (≥7 stars), while 22 studies (16–19, 21, 23–30, 32–35, 37, 39–41, 43) were classified as moderate quality (5–6 stars).

Most studies performed well in the selection and outcome domains, particularly those that employed validated survey instruments (e.g., HSOPSC, EQS-H, ED-CAHPS) and achieved adequate response rates. In contrast, performance in the comparability domain was generally weaker, as adjustment for potential confounding variables was rarely undertaken. Moderate-quality ratings were commonly attributable to small or convenience-based samples, reliance on self-reported data, and the predominantly single-centre design of included studies, which limited generalisability.

By comparison, studies rated as high quality were typically characterised by larger or multicentre samples, higher response rates, use of validated outcome measures, and more rigorous statistical reporting. Overall, the risk of bias appraisal suggests that while the evidence base is of acceptable methodological quality, findings from smaller, single-centre, or non-adjusted studies should be interpreted with caution. A summary of NOS scores and quality classifications for each study is presented in Table 3.

**Table 3:** The NOS appraisal of the studies included in this systematic review.

Study	Selection (0–5)	Comparability (0–2)	Outcome (0–3)	Total (0–10)	Quality
Abass et al. (16)	4	0	2	6	Moderate
Alhumud et al. (17)	3	0	2	5	Moderate
Alnasser et al. (19)	4	0	2	6	Moderate
Aljaffary et al. (20)	5	1	3	9	High
Bokhary et al. (18)	3	0	2	5	Moderate
Aljadhey et al. (21)	4	0	2	6	Moderate
Traiki et al. (41)	4	0	2	6	Moderate
Abualenain et al. (39)	4	0	2	6	Moderate
Albarrak et al. (22)	4	1	3	8	High
Almalky et al. (23)	3	0	2	5	Moderate
Al-Abbadi et al. (24)	4	0	2	6	Moderate
Alswat et al. (42)	5	1	3	9	High
Albishi et al. (25)	3	0	2	5	Moderate
Aljuaid et al. (34)	4	0	2	6	Moderate
Aljohaney et al. (31)	4	0	3	7	High
Alnasser et al. (36)	5	1	3	9	High
AlRuthia et al. (32)	4	0	2	6	Moderate
Memon (33)	3	0	2	5	Moderate
Balhaddad et al. (26)	3	0	2	5	Moderate
Attar et al. (27)	3	0	2	5	Moderate
Alshareef et al. (35)	4	0	2	6	Moderate
Arishi et al. (37)	3	0	2	5	Moderate
Alzakri et al. (28)	4	0	2	6	Moderate
AlThubaity and Shalby (29)	3	0	2	5	Moderate
Mulla et al. (38)	4	1	3	8	High
AlSadah et al. (40)	4	0	2	6	Moderate
Badheeb et al. (43)	3	0	2	5	Moderate
Alsohime et al. (30)	4	0	2	6	Moderate

## 4 Discussion

This systematic review provides a comprehensive and updated synthesis of evidence on the quality of care in Saudi university-affiliated hospitals, drawing on 28 studies published between 2016 and 2025 and examining all six IOM quality domains. Overall, the findings demonstrate meaningful progress in selected areas, particularly patient-centredness, effectiveness of targeted interventions, and efficiency gains through innovative service models. However, persistent challenges remain in safety culture, timeliness of care, and equity of access, highlighting important gaps that warrant focused attention in the context of Saudi Arabia's ongoing health system transformation under Vision 2030.

Patient-centredness emerged as the most extensively examined domain, with multiple studies reporting high levels of satisfaction related to provider communication, respect, and interpersonal interactions (16, 23, 41). These findings are consistent with recent national and regional reviews, including Alasiri et al. (44), which reported high satisfaction levels in Saudi academic hospitals but identified ongoing concerns related to access, waiting times, and provider availability. Compared with the earlier systematic review by Aljuaid et al. (11), which highlighted limited patient engagement and disease-focused care, the more recent evidence suggests gradual improvement in communication practices and patient involvement. Nevertheless, systemic barriers such as referral delays, long waiting times (16, 31), and restricted access to specialised services (28) continue to undermine patient-centred care, indicating that improvements in interpersonal quality have not been matched by equivalent gains in service organisation and accessibility.

In terms of effectiveness, this review identified strong outcomes in selected clinical areas, including paediatric services (22) and perioperative pain management (40). However, challenges persist in chronic disease management, as patients with psoriatic arthritis continue to experience impaired quality of life due to disease activity, fatigue, and comorbidities (27). Rehabilitation services also faced adherence challenges related to pain and geographic distance, although provider support and goal setting were identified as effective facilitators (28). Importantly, innovative care models such as hypofractionated radiotherapy (38) and telemedicine follow-up services (35) demonstrated both high patient satisfaction and improved effectiveness of care delivery. These findings align with Aljuaid et al. (11), who previously reported suboptimal adherence to clinical guidelines and gaps in palliative and chronic care, suggesting that although challenges remain, service innovations are expanding the scope of effective care delivery.

Timeliness was one of the most consistently problematic domains across studies, with prolonged waiting times, delayed discharge processes, and extended emergency department stays commonly reported (32, 43). These delays were strongly associated with lower patient satisfaction (16, 31) and reflect broader system-level inefficiencies previously identified in Saudi and GCC healthcare systems (1, 2). Importantly, several studies (23, 35) demonstrated that digital and remote care models such as telepsychiatry and tele-rhinology can mitigate delays and improve timely access, underscoring the potential of technology-enabled solutions to address longstanding timeliness challenges in academic hospitals. These results are consistent with the review by Aljuaid et al. (11), which highlighted limited evidence on timeliness, and extend it by demonstrating more robust evidence of systemic delays alongside emerging digital solutions.

Findings related to efficiency revealed a mixed picture. On the one hand, substantial inefficiencies were evident, including high proportions of non-urgent emergency department visits (36), prolonged discharge workflows (32), and underutilisation of incident reporting systems (33). On the other hand, academic hospitals appeared to be at the forefront of adopting innovative service delivery models that enhance efficiency, including telemedicine and streamlined radiotherapy protocols (23, 35). These findings resonate with those of Alluhaymid and Alabdrabalnabi (45), who reported widespread inefficiencies in public hospitals, but they also suggest that academic hospitals may be more actively adopting innovative care models to mitigate such inefficiencies.

Safety-related findings were prominent and largely consistent with previous national and regional literature (1, 2). Persistent concerns included medication errors (33, 34, 37), limited adherence to infection prevention practices (25), staffing shortages, and weak non-punitive safety cultures (20, 42). At the same time, strengths in organisational learning, teamwork (20), and the adoption of technologies such as point-of-care testing (39) and electronic health records (30) supported safer practices. These findings extend those of Aljuaid et al. (11), who also highlighted weaknesses in safety culture, and align with those of Alluhaymid and Alabdrabalnabi (45), who reported punitive cultures and staffing issues as barriers to safety in public hospitals.

Equity was the least examined domain, reflecting a significant evidence gap. The limited available studies (27, 38) highlighted demographic and geographic disparities in health outcomes and access to specialised services, particularly for patients travelling long distances to tertiary care centres. These findings are consistent with broader system-level reviews showing persistent inequities linked to geography and socioeconomic status in Saudi Arabia (1, 11, 44). The scarcity of equity-focused research within academic hospitals represents a critical gap, particularly given Vision 2030's emphasis on equitable access and inclusive healthcare delivery.

Finally, the risk of bias appraisal indicated that six studies (20, 22, 31, 36, 38, 42) were rated high quality, while the majority (*n* = 22) were of moderate quality. Common limitations included small sample sizes, single-centre settings, convenience sampling, and limited adjustment for confounding factors. These issues mirror the methodological concerns highlighted in all three previous systematic reviews (44, 45), underscoring the need for stronger study designs, multicentre collaborations, and standardised outcome measures in future research.

## 5 Implications for policy, education, and practice

The findings of this review have important implications for policy, education, and quality improvement in Saudi academic hospitals. From a policy perspective, persistent challenges in timeliness, safety, and equity suggest the need for targeted performance monitoring frameworks aligned with IOM domains and Vision 2030 objectives. Academic hospitals should play a central role in piloting quality improvement initiatives, digital health solutions, and integrated care models that can be scaled across the health system.

From an educational standpoint, the dual service education mandate of academic hospitals positions them uniquely to embed quality and safety competencies within medical and health profession training. Strengthening curricula on patient safety, quality improvement, and patient-centred care may contribute to sustainable improvements in care delivery. At the organisational level, investment in leadership development, workforce capacity, and non-punitive safety cultures is essential to translating innovation into consistent quality gains.

## 6 Limitations

This review has several limitations that should be considered when interpreting the findings. First, study screening and data extraction were led by a single author. However, an independent reviewer verified a subset of records, and any uncertainties or disagreements were resolved through discussion with a senior researcher. Although this approach enhanced rigour, the absence of fully independent dual screening across all stages may have introduced a degree of selection or interpretation bias. Second, most included studies were of moderate methodological quality and were commonly characterised by single-centre designs, convenience sampling, and limited adjustment for confounding variables, which may restrict the generalisability of findings. Third, evidence related to equity was scarce, limiting the ability to draw robust conclusions for this domain. Despite these limitations, this review provides the most comprehensive and up-to-date synthesis of evidence on the quality of care in Saudi university-affiliated hospitals.

## 7 Contribution of this review

This review makes several novel contributions to the literature. It nearly triples the evidence base compared with the 2016 Saudi-focused review (45), incorporates all six IOM quality domains, and uniquely focuses on university-affiliated hospitals during the post-Vision 2030 reform period. By integrating institutional-level evidence with national health transformation priorities, this review provides a critical bridge between quality assessment, policy reform, and academic healthcare practice in Saudi Arabia.

## 8 Conclusion

This systematic review provides the most comprehensive assessment to date of the quality of care in Saudi university-affiliated hospitals, synthesising evidence from 28 studies published between 2016 and 2025 across all six IOM quality domains. The findings highlight important strengths in patient-centredness, effectiveness of targeted services, and efficiency gains driven by innovative care models, particularly telemedicine and streamlined treatment pathways. However, persistent challenges in timeliness, safety culture, and equity of access underscore the need for targeted quality improvement efforts.

To support Saudi Arabia's Vision 2030 health transformation agenda, academic hospitals must strengthen performance monitoring, invest in safety and workforce development, and expand the use of digital health solutions to reduce delays and improve equitable access. As centres of clinical care, education, and research, university-affiliated hospitals are well positioned to lead quality improvement initiatives that can inform national policy and practice. Future research should prioritise multicentre studies, robust adjustment for confounding factors, and greater focus on equity to support evidence-based decision-making and sustainable quality improvement across the Saudi health system.

## Data Availability

The original contributions presented in the study are included in the article/Supplementary Material; further inquiries can be directed to the corresponding author.
